# Heart Rate Variability Analyses in Parkinson’s Disease: A Systematic Review and Meta-Analysis

**DOI:** 10.3390/brainsci11080959

**Published:** 2021-07-21

**Authors:** Konstantin G. Heimrich, Thomas Lehmann, Peter Schlattmann, Tino Prell

**Affiliations:** 1 Department of Neurology, Jena University Hospital, 07747 Jena, Germany; tino.prell@med.uni-jena.de; 2 Institute of Medical Statistics, Computer and Data Sciences, Jena University Hospital, 07743 Jena, Germany; thomas.lehmann@med.uni-jena.de (T.L.); peter.schlattmann@med.uni-jena.de (P.S.); 3 Center for Healthy Ageing, Jena University Hospital, 07747 Jena, Germany

**Keywords:** Parkinson’s disease, heart rate variability, vagus nerve, autonomic nervous system, autonomic dysfunction

## Abstract

Recent evidence suggests that the vagus nerve and autonomic dysfunction play an important role in the pathogenesis of Parkinson’s disease. Using heart rate variability analysis, the autonomic modulation of cardiac activity can be investigated. This meta-analysis aims to assess if analysis of heart rate variability may indicate decreased parasympathetic tone in patients with Parkinson’s disease. The MEDLINE, EMBASE and Cochrane Central databases were searched on 31 December 2020. Studies were included if they: (1) were published in English, (2) analyzed idiopathic Parkinson’s disease and healthy adult controls, and (3) reported at least one frequency- or time-domain heart rate variability analysis parameter, which represents parasympathetic regulation. We included 47 studies with 2772 subjects. Random-effects meta-analyses revealed significantly decreased effect sizes in Parkinson patients for the high-frequency spectral component (HFms^2^) and the short-term measurement of the root mean square of successive normal-to-normal interval differences (RMSSD). However, heterogeneity was high, and there was evidence for publication bias regarding HFms^2^. There is some evidence that a more advanced disease leads to an impaired parasympathetic regulation. In conclusion, short-term measurement of RMSSD is a reliable parameter to assess parasympathetically impaired cardiac modulation in Parkinson patients. The measurement should be performed with a predefined respiratory rate.

## 1. Introduction

Parkinson’s disease (PD) is one of the most common neurodegenerative disorders. As a multisystem disorder, PD is characterized by motor symptoms and a plethora of nonmotor symptoms [1,2,3]. An increasing number of studies indicate that these are at least partly caused by changes in the gut-brain axis [4], dysbiosis with local inflammation [5], and finally alpha-synuclein accumulation in the enteric nervous system [6]. These pathological proteins may spread via the vagus nerve to the central nervous system [7]. The vagus nerve is the main parasympathetic branch of the autonomic nervous system. Additionally, central dopaminergic cell death can favor central inflammatory processes that affect the vagus nerve directly [8]. An altered function of the autonomic nervous system can cause various nonmotor symptoms, accordingly, many PD patients have symptoms of dysautonomia [9]. Frequently, patients complain of hypersalivation, swallowing difficulties, delayed gastric emptying, constipation, or orthostatic hypotension [10]. 

Assessing parasympathetic regulation in PD is a promising way to assess symptoms and to improve our knowledge of the course of the disease. Similarly, improved dysautonomia testing may be of additional value in differentiating PD and atypical parkinsonian syndromes. Heart rate variability (HRV) analysis is a simple method to estimate the overall tone of the autonomic nervous system [11]. Whereas heart rate quantifies the number of heartbeats per minute, HRV refers to the fluctuation in the time between successive heartbeats. It reflects the sympathetic and parasympathetic modulation of cardiac activity. In cardiology, there are recommendations for HRV analysis for standards of measurement, physiological interpretation and clinical use [12]. Recommendations for clinical use in patients with neurodegenerative diseases do not exist. In the literature, there are many different approaches to studying HRV in PD.

In general, a distinction can be made in (1) frequency-domain indices, (2) time-domain indices, and (3) nonlinear measurements [11]. Frequency-domain indices are used to describe the power spectral density as a function of frequency using mathematical algorithms. Short-term recordings can distinguish high-frequency (HF), low-frequency and very-low-frequency components. Long-term recordings can additionally distinguish ultra-low-frequency components. The HF component is generated mainly by parasympathetic modulation [11]. Time-domain indices quantify HRV observed during monitoring periods ranging from shorter than 1 minute to longer than 24 h. To describe parasympathetic regulation, there are two established parameters. The root mean square of successive normal-to-normal interval differences (RMSSD) in ms reflects the beat-to-beat variance, and the percentage of adjacent normal heartbeat intervals that differ from each other by more than 50 ms (pNN50). 

In addition to these mentioned statistical measurements, time-domain recordings can also be converted into nonlinear geometric patterns. However, these geometric patterns mainly describe the total variance and not parasympathetic activity [11], and will therefore not be considered in this review. In this systematic review of parasympathetic modulation in PD, we will focus on the HF spectral component, RMSSD, and pNN50. Lower values of HF, RMSSD, and pNN50 are an indication of reduced parasympathetic regulation.

The objective of our study is to systematically review the literature if a frequency- or time-domain analysis of the HRV may indicate a decreased parasympathetic tone in patients with PD. Our secondary aim is to determine the most suitable HRV method for clinical use in PD.

## 2. Materials and Methods

### 2.1. Search Strategy and Eligibility Criteria

We conducted our systematic review and meta-analysis in accordance with the Preferred Reporting Items for Systematic Reviews and Meta-Analyses (PRISMA) [13]. This study does not constitute human subject research. Therefore, there was no need for local ethics committee approval. The search strategy was created in consultation with a medical statistician with expertise in systematic review searching. Three electronic databases (MEDLINE, EMBASE, and Cochrane Central) were screened from inception to 31 December 2020, without any restriction on the year of the study. We searched MEDLINE with the Medical Subject Headings “Parkinson disease” (Mesh) AND “heart rate” (Mesh) and EMBASE with the subject headings “Parkinson disease” AND “heart rate variability” and limited the records to humans, English, and articles. Our search in Cochrane Central was conducted using the keywords “Parkinson” AND “heart rate variability” and allowing word variations. The reference lists of included studies or relevant reviews identified through the search were searched to identify any studies missed in the initial search to ensure literature saturation. Only full-text articles were considered for analysis. References were managed using EndNote (version X8; Clarivate Analytics). Titles and abstracts were screened by one reviewer (K.G.H.) and controlled by a second reviewer (T.P.) if the study was excluded.

Studies needed to be original research. We included studies with an observational design, randomized controlled trials, controlled (nonrandomized) clinical trials, and interrupted time series studies if there was at least one reported data point before the intervention. Review articles, book reviews, short communications, and correspondences were excluded.

Two reviewers assessed the full-text articles for eligibility (K.G.H. and T.P.). Different assessments were resolved by consensus. Articles were included if they: (1) were published in English, (2) reported patients with idiopathic PD as a primary study group and healthy adult controls as a secondary study group, and (3) reported at least one HRV parameter, which represents parasympathetic activity (HF spectral component, RMSSD, or pNN50).

### 2.2. Data Extraction

The data extraction form was developed in Microsoft Excel (version 2016; Microsoft Corporation). The data extraction was performed by one member of the research team (K.G.H.) and checked by a second (T.P.). The following data were extracted: study information (author, title, journal, and year of publication), study characteristics (e.g., study setting), participant characteristics (e.g., age, sex, disease severity, and disease duration), condition information (i.e., data sources, total number of participants, and exclusion criteria), and HRV parameters (HF, RMSSD, and pNN50). Data on participant characteristics and HRV parameters were extracted and, if possible, grouped to achieve better evaluability. The results of substudies were extracted separately in order to allow subsequent subgroup analysis based on different subgroup characteristics. If there were incomplete data reported in the publication, we searched supplementary information and documents to locate missing data.

### 2.3. Statistical Analyses

For each study population, the main characteristics were reported as mean ± SD (standard deviation) for continuous variables and the number (%) for categorical variables. If not reported, the sample mean and SD were synthetized and estimated from the sample size, median, range and/or interquartile range [14]. Random-effects meta-analyses by means of the Hartung-Knapp-Sidik-Jonkman method were conducted. If substudies were reported, the raw mean of each study was calculated to avoid an overestimation of the impact. Data were analyzed separately for each HRV parameter. Hedges’ g was used as a measure of standardized mean differences (SMD). 

We estimated the significance and degree of heterogeneity in the study results by Cochrane’s Q, I^2^-statistics, and Tau^2^-statistics. To evaluate the heterogeneity, we searched for outliers. Additionally, we performed influence analyses by means of the leave-one-out method and GOSH plot analyses.

We conducted random-effects subgroup analyses to assess measurement time-dependent between-subgroup differences. Additionally, subgroup analyses were carried out between normal and deep breathing to assess the impact of respiration on HRV. Deep breathing with a predefined respiratory rate standardized the respiration-driven acceleration and deceleration of the heart rate [15].

We considered studies that performed both normal and deep breathing in the same population to assess comparability.

Random-effects meta-regression analyses were performed to examine the relationship between HRV parameters and relevant covariates. We checked for multicollinearity. We considered predictors to be highly correlated if r > 0.8.

Publication bias was assessed through Egger’s Test. A *p*-value of Egger’s test < 0.05 is significant, which means that there is substantial asymmetry in the funnel plot. When Egger’s test was significant, the trim-and-fill procedure was used to adjust for publication bias. 

We did not perform a detailed quality assessment of each included study. First, because mainly no intervention effect was analyzed and second, because the statement of the initiative to strengthening the reporting of observational studies in epidemiology (STROBE statement) should not be used as a tool to assess the methodological quality of cohort studies [16].

For statistical analyses, R (The R Foundation for Statistical Computing, version 3.6.3) and RStudio (PBC, version 1.3.1093) were used with meta (General Package for Meta-Analysis, version 4.15-1), metafor (Meta-Analysis Package for R, version 2.4-0), Hmisc (Harrell Miscellaneous, version 4.4-1) and dmetar (Companion R package for the guide ‘Doing Meta-Analysis in R’, version 0.0.90000) [17].

## 3. Results

### 3.1. Selection and Study Population

**Figure 1:**
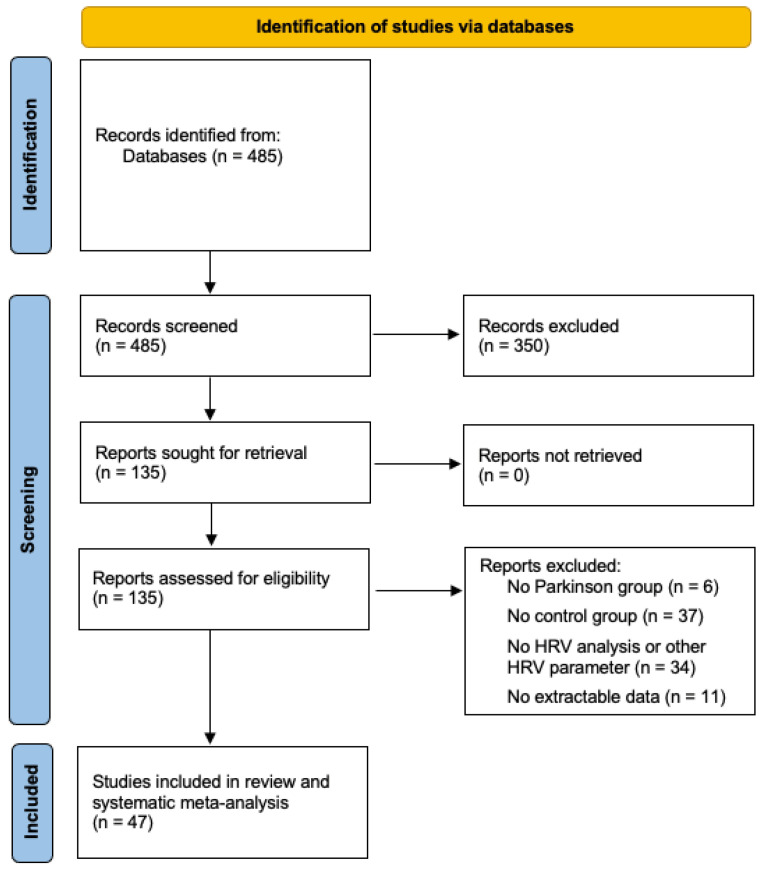
PRISMA flow diagram. HRV: heart rate variability.

We screened 485 unique citations. Of these, 135 were assessed for eligibility, and 47 studies were included in this systematic review and meta-analysis [18,19,20,21,22,23,24,25,26,27,28,29,30,31,32,33,34,35,36,37,38,39,40,41,42,43,44,45,46,47,48,49,50,51,52,53,54,55,56,57,58,59,60,61,62,63,64]. For further details on study selection, see Figure 1. The study population includes 2772 subjects. Of these, 1566 had PD, and 1206 were healthy controls. The summarized findings of the population are shown in Table 1.

### 3.2. Study Characteristics

All included studies used HRV analysis to assess autonomic function in PD patients compared to controls. Most studies focused on the measurement of HRV under particular conditions regarding time, duration, and method, or they intended to describe a relation between HRV and the severity of PD. An overview of the included studies can be found in Table A1 in the Appendix A. 

Patients with a confirmed PD diagnosis according to the United Kingdom Parkinson’s Disease Society Brain Bank clinical diagnostic criteria or the Movement Disorder Society (MDS) clinical diagnostic criteria were included. Exclusion criteria varied. In particular, these are cardiovascular diseases, endocrinological diseases (especially diabetes), peripheral or central neurological disorders (except PD), liver, kidney or lung diseases, and medication known to affect the autonomic nervous system. Eight studies stated that any medical disorder known to affect the autonomic nervous system was excluded [19,21,22,30,36,48,53,58]. Cardiovascular diseases, diabetes, and selected drugs were excluded in 13 studies [19,22,28,31,32,33,34,35,43,45,55,58,63]. No exclusion criteria were reported in five studies [24,42,44,52,59].

The duration of the measurements varied from 1 minute to 24 h. Because there is no uniformly accepted limit, we categorized the study into short-term (shorter than one hour) and long-term (longer than one hour) measurements. Short-term measurements were carried out in 34 studies, with a range from 1–20 min. Long-term measurements were carried out in 16 studies, with a range from 6–24 h. Short-term measurements were mainly conducted during daytime and long-term measurements during day- and nighttime.

### 3.3. Random-Effects Meta-Analyses

We conducted a random-effects meta-analysis for each HRV parameter. The results are shown in Table 2(A), raw analyses.

#### 3.3.1. Frequency-Domain Parameters of HRV

Mainly, the HF components of the power spectral density are either measured in absolute values (ms^2^) or in normalized units (nu). These data will be analyzed below. Studies, which reported data only in the rarely used units ln [31,42,62], log [60], ms/Hz [57], and *10^−1^s^2^/Hz [32] were not considered in the following analyses of HF. 

**Figure 2:**
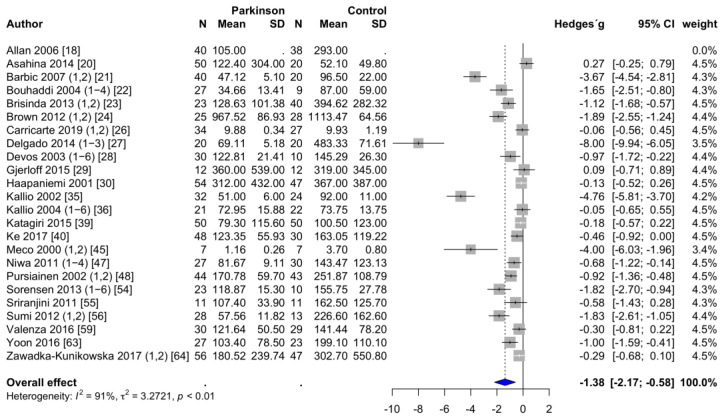
Forest plot HFms^2^, random-effects meta-analysis. HFms^2^: high-frequency components of the power spectral density in ms^2^; N: number; SD: standard deviation; CI: confidence interval. Rhombus indicates meta-analytically pooled estimate of the 95% confidence interval. The numbers after the authors indicate the included substudies according to the information provided in Appendix A, Table A1.

**HFms^2^.** HFms^2^ was reported in 24 studies [18,20,21,22,23,24,26,27,28,29,30,35,36,39,40,45,47,48,54,55,56,59,63,64]. The mean HFms^2^ was 145.2 ± 41.1 ms^2^ in PD patients and 219.4 ± 48.8 ms^2^ in the healthy control group. Two studies had to be excluded [33,58] because the powers of ten differed by a factor of three to four. Accordingly, the values of these two studies were not considered plausible. PD patients had significantly lower HFms^2^ values than healthy controls. The forest plot of the effect sizes for HFms^2^ is shown in Figure 2. Egger’s test revealed evidence of publication bias (*p* < 0.001). Trim-and-fill procedure indicated seven missing studies. The adjusted estimate of the effect size changed substantially, leading to a nonsignificant effect (SMD = −0.46; 95% CI = −1.45 to 0.52; *p* = 0.344; I^2^ = 94%).

**Figure 3:**
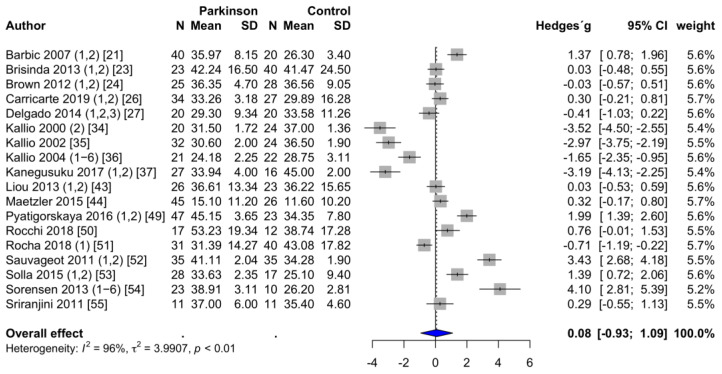
Forest plot HFnu, random-effects meta-analysis. HFnu: High-frequency components of the power spectral density in normalized units.

**HFnu**. HFnu was reported in 18 studies [21,23,24,26,27,34,35,36,37,43,44,49,50,51,52,53,54,55]. The mean HFnu was 34.7 ± 1.8 in PD patients and 33.2 ± 1.9 in the healthy control group. One study had to be excluded because the powers of ten differed by a factor of two [58]. The values of this study were therefore considered to be not plausible. Contrary to the calculation of HFms^2^, no significant difference was determined regarding HFnu. The forest plot can be seen in Figure 3. Egger’s test revealed no evidence of publication bias (*p* = 0.772). 

In nine studies, both HFms^2^ and HFnu were assessed [21,23,24,26,27,35,36,54,55] with no significant linear correlation (r_p_ = 0.21, *p* = 0.583).

#### 3.3.2. Time-Domain Parameters of HRV

**Figure 4:**
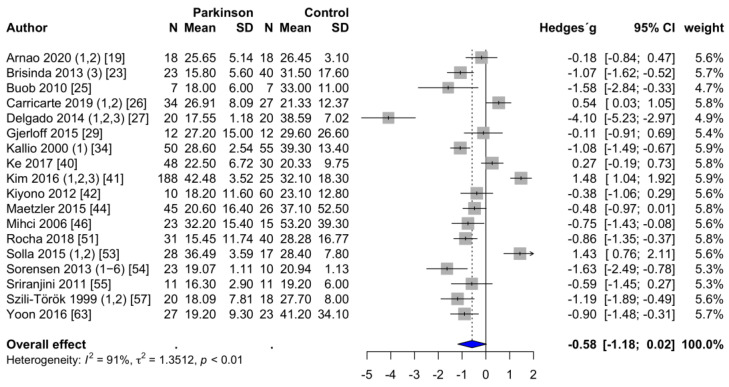
Forest plot RMSSD, random-effects meta-analysis. RMSSD: Root mean square of successive normal-to-normal interval differences.

**RMSSD**. The RMSSD in ms was reported in 18 studies [19,23,25,26,27,29,34,40,41,42,44,46,51,53,54,55,57,63]. Two studies assessed RMSSD but had to be excluded. Of them, one study reported the results logarithmically [60]. The other study was excluded because the powers of ten differed by a factor of three, and the values were therefore considered to be not plausible [61]. In PD patients, the mean RMSSD was 23.4 ± 1.9 ms, compared to 28.9 ± 1.8 ms in the healthy control group. However, these differences are statistically not significant (*p* = 0.059). This finding is shown in the forest plot in Figure 4. Egger’s test revealed no evidence of publication bias (*p* = 0.096).

**Figure 5:**
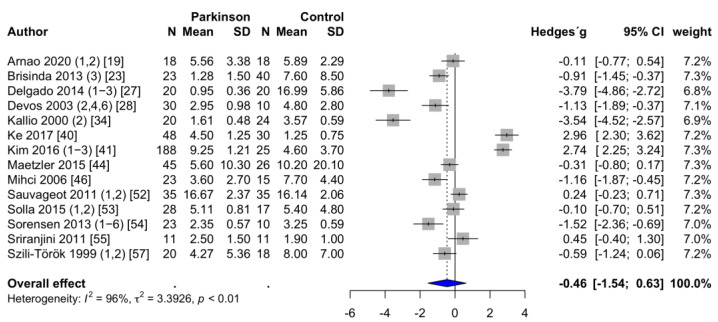
Forest plot pNN50, random-effects meta-analysis. pNN50: Percentage of adjacent normal heartbeat intervals that differ from each other by more than 50 ms.

**pNN50**. The pNN50 was reported in 14 studies [19,23,27,28,34,40,41,44,46,52,53,54,55,57]. The mean pNN50 was 4.7 ± 1.1% in PD patients and 6.8 ± 1.3% in the healthy control group. These differences are statistically not significant (*p* = 0.378). According to Egger’s test, there was evidence of publication bias (*p* = 0.043). Adding the four missing studies that the trim-and-fill procedure identified, the adjusted estimate of the effect size did not change substantially. There was still no significant effect size (SMD = 0.36; 95% CI = −0.82 to 1.53; *p* = 0.532; I^2^ = 97%). The forest plot can be seen in Figure 5.

### 3.4. Between-Study Heterogeneity

High values for I^2^ were found for all HRV parameters. We evaluated heterogeneity by performing outlier analysis, influence analysis, and GOSH plot analysis for each HRV parameter. Then, we excluded studies that were identified with a potentially high risk of bias. Again, a random-effects meta-analysis was conducted. The results are shown in Table 2(B), heterogeneity analyses. Taken together, in PD patients, there were still significantly lower values for HFms^2^. Additionally, there were significant effect sizes regarding RMSSD and pNN50. I^2^ decreased considerably. However, there was still a moderate degree of heterogeneity, indicating different patient populations, interventions, or measurement methods.

### 3.5. Subgroup Analyses

**Figure 6:**
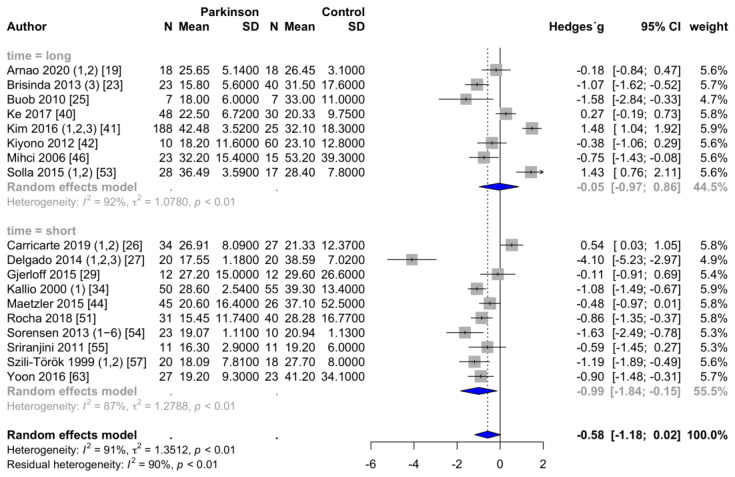
Forest plot RMSSD, subgroup measurement time.

PD patients had significantly lower HFms^2^ values both during short-term and long-term measurements than healthy controls (short-term, SMD = −1.63; 95% CI = −2.82 to −0.44, *p* = 0.011, I^2^ = 84%; long-term, SMD = −0.85; 95% CI = −1.68 to −0.01, *p* = 0.047, I^2^ = 73%). There were no significant between-group differences (Q = 1.41, *p* = 0.235). Regarding HFnu, neither short-term nor long-term measurements revealed a significant effect size between PD patients and controls (short-term, SMD = −0.29, 95% CI = −1.51 to 0.93, *p* = 0.615, I^2^ = 95%; long-term, SMD = 1.04, 95% CI = −1.36 to 3.43, *p* = 0.296, I^2^ = 97%). RMSSD is significantly reduced in the short-term setting but not in the long-term setting (short-term, SMD = −0.99, 95% CI= −1.84 to −0.15, *p* = 0.026, I^2^ = 87%; long-term, SMD = −0.05, 95% CI = −0.97 to 0.86, *p* = 0.895, I^2^ = 92%), which is shown in the forest plot in Figure 6. There were no significant effect sizes regarding pNN50 during short-term and long-term measurements (short-term, SMD = −1.52, 95% CI = −3.35 to 0.31, *p* = 0.086, I^2^ = 93%; long-term, SMD = 0.32, 95% CI = −1.05 to 1.70, *p* = 0.596, I^2^ = 97%).

We also performed a subgroup analysis to assess differences between normal and deep breathing. Deep breathing was applied in five studies [24,27,43,44,61]. Of them, two studies analyzed HFms^2^ [24,27]. There were no significant differences in either the normal breathing or the deep breathing subgroup between PD patients and controls (normal breathing, SMD = −2.26; 95% CI = −6.52 to 2.01, *p* = 0.151, I^2^ = 95%; deep breathing, SMD = −1.77, 95% CI = −21.70 to 18.15, *p* = 0.461, I^2^ = 97%). Regarding HFnu we considered three studies [24,27,43]. There were no significant differences in these two subgroups between PD patients and controls (normal breathing, SMD = −0.60; 95% CI = −1.82 to 0.61, *p* = 0.212, I^2^ = 83%; deep breathing, SMD = 0.26, 95% CI = −1.11 to 1.64, *p* = 0.496, I^2^ = 71%). Only one study assessed the time-domain HRV parameters RMSSD and pNN50 during normal and deep breathing [27]. According to the study results both normal breathing and deep breathing revealed significantly lower values in PD patients than in controls (RMSSD: normal breathing, SMD = −3.76, 95% CI = −6.85 to −0.66, *p* = 0.041; deep breathing, SMD = −4.28, 95% CI = −5.45 to −3.11, *p* < 0.001; pNN50: normal breathing, SMD = −2.73, 95% CI = −4.67 to −0.78, *p* = 0.036; deep breathing, SMD = −4.85, 95% CI = −6.13 to −3.57, *p* < 0.001).

### 3.6. Meta-Regression

First, we considered patient age, sex, disease duration and severity of disease determined by Hoehn and Yahr stage, Unified Parkinson’s Disease Rating Scale (UPDRS) score and UPDRSIII subscore separately. A longer disease duration significantly contributed to lower HFms^2^ (*p* = 0.033), but only in the long-term measurement. A higher Hoehn and Yahr stage was associated with lower RMSSD (*p* = 0.0274), but only in the short-term measurement. 

Second, we conducted multiple meta-regression analyses. The UPDRS score and UPDRSIII subscore are with a substantial degree of accuracy significantly linearly predicted from the Hoehn and Yahr stage (r_p_ = 0.92 and r_p_ = 0.81, respectively). Accordingly, as covariates, we included patient age, sex, disease duration and Hoehn and Yahr stage. Multiple meta-regressions revealed no significant contribution of any covariate to one of the HRV parameters.

## 4. Discussion

PD patients exhibited a strong decrease in parasympathetically modulated HRV parameters compared to healthy controls. In our meta-analysis, PD was associated with lower values of HFms^2^ and lower values of RMSSD during short-term measurements. No decrease was seen regarding HFnu. The normalized unit of HF is the relative value of HF in proportion to the total power minus the very-low-frequency component. Total power includes all frequencies and depends on the regulation of both branches of the autonomic nervous system. Therefore, normalization tends to minimize the parasympathetic influence. Accordingly, it must be emphasized at this point that a determination of HFnu does not allow any valid statement on parasympathetic modulation, because it reflects the overall autonomic tone.

As shown in the meta-regression analyses, there is some evidence that more advanced PD leads to an increasing reduction in these parasympathetically modulated HRV parameters. This outcome corresponds to the assumption of increasing damage to the vagus nerve in the course of the disease [61,65]. Especially regarding HFms^2^, disease duration significantly contributes to the effect size. Additionally, the Hoehn and Yahr stage significantly contributed to the effect size of RMSSD. However, these correlations could not be consistently ascertained for all HRV parameters. Clinical parameters regarding the duration and severity of the disease have not been reported in many studies. The UPDRSIII subscore was only reported in 16 of 47 studies. Only two studies used the MDS-UPDRSIII subscore [29,61], although it was published in 2007 and should be used preferably [66]. Insufficient data reporting precluded studies from the meta-regression model.

It is known that age and sex have an important impact on HRV [67,68,69,70,71]. Aging causes neuronal and structural modifications of the cardiorespiratory system leading to a decreased vagal tone [68,70]. The influence of patient age could not be confirmed by our results. Generally, it is assumed that females show greater vagal tone than males [71]. This differential autonomic tone indicates age- and sex-related predisposition to cardiovascular diseases. We could not find evidence that in PD, HRV parameters depended significantly on sex. This result may be due to progressive sex-independent vagal damage in the course of PD.

Heterogeneity of the studies was high. Besides a limited number of studies, this indicates different patient populations and diverse study settings. Especially regarding frequency-domain measurements, many studies had to be classified as outliers or influence studies. High heterogeneity makes valid regression analysis difficult and complicates the investigation of relevant covariates regarding disease severity. This issue could be improved through a standardized basic measurement in addition to a study-specific measurement and through reporting of defined clinical parameters. This would allow for improved subgroup analysis.

The importance of a subgroup analysis becomes clear when considering the measurement time. Considering measurement time, it is evident that RMSSD is significantly reduced in PD patients only during short-term measurements. Theoretically, a 24-h recording seems to be advantageous because the total variance of HRV increases. However, the prerequisite for this would be that the mechanisms responsible for heart rate modulation remain unchanged during the recording period to ensure a largely stable heart rate and respiratory rate. Otherwise, the results of HRV analysis may be more due to external influences than to autonomic regulation. In particular, physiological mechanisms of heart rate modulation cannot be considered stationary over a 24-h period [72]. Therefore, short-term measurements under stationary conditions, especially regarding physical activity, position, and temperature, seem to be advantageous, which is supported by our meta-analysis.

To ensure literature saturation patients with a leucine-rich repeat kinase 2 mutation (LRRK2) associated PD were not primarily excluded. Additionally, most of the patients were not explicitly genetically tested and therefore it cannot be excluded that LRRK2-associated PD patients were included in other study populations. Autonomic dysfunctions are associated with genetic forms of synucleinopathies like LRRK2-associated PD [73]. Within this meta-analysis we identified two studies, which examine HRV in LRRK2-associated PD patients [26,60]. These studies showed higher parasympathetically modulated HRV parameters in LRRK2-associated PD patients compared to idiopathic PD. Therefore, it can be assumed that the described effect sizes are rather underestimated.

It is well known that respiration has a major impact on heart rate [74]. The heart rate increases during inspiration and decreases during expiration, which is called respiratory sinus arrhythmia [15]. Measurements conducted with a predefined deep breathing rate enable HRV analyses to reflect the vagal tone rather than differences in respiration [72]. The obtained results of our subgroup analyses regarding normal and deep breathing should be interpreted with caution. The results are based on the values of a very limited number of studies. Especially regarding RMSSD and pNN50, the results are obtained from just one study [27]. Therefore, it is strongly recommended to perform HRV analysis with a predefined respiratory rate. Otherwise, the significance of the analysis is very limited. This should be considered in further studies. 

In addition to heterogeneity and meta-regression analyses, the results have to be evaluated under consideration of publication bias. Rather unexpectedly, there was no significant adjusted effect size regarding HFms^2^. However, a subgroup analyses revealed that there is evidence of publication bias only in the short-term measurement of HFms^2^, and not in the long-term measurement.

## 5. Conclusions

HRV analysis is an easy tool to measure vagal influence on the heart rate, and indirectly, to assess autonomic dysfunction in PD. PD patients showed decreased parasympathetically modulated HRV parameters compared to healthy controls. This finding is in line with the assumption of vagal damage in the course of the disease. After considering heterogeneity analysis, subgroup analysis and evaluation of publication bias, we recommend establishing a short-term measurement of RMSSD as a basic measurement of HRV in future studies. At the very least, this parameter should be reported as a basic measurement in addition to study-specific measurements. To standardize the impact of respiration, the measurement should be performed with a predefined respiratory rate of around six respiratory cycles per minute. To enable further assessment of disease progression and in the attempt to distinguish PD and atypical parkinsonian syndromes, we recommend specifying defined clinical parameters of the patient collective. In particular, patient age, sex, disease duration and severity of disease determined by Hoehn and Yahr stage are easy to collect even for non-neurologists. A specification of the MDS-UPDRSIII would further improve the quality of future studies.

## Figures and Tables

**Table 1:** Study population, characteristics.

Characteristics	PD Patients (*n* = 1566)	Controls (*n* = 1206)
Gender (male, %)	60.8	55.3
Age (years)	65.0 ± 0.6	62.6 ± 1.0
Disease duration (years)	5.8 ± 0.5	-
Hoehn and Yahr stage	2.2 ± 0.1	-
UPDRS	32.3 ± 3.6	-
UPDRSIII	21.4 ± 2.2	-

Values are given as the mean ± SD, unless otherwise indicated; PD: Parkinson’s disease; UPDRS: Unified Parkinson’s Disease Rating Scale, total score; UPDRSIII: Unified Parkinson’s Disease Rating Scale, subscore III motor part. Data of the Movement Disorder Society revised version of the UPDRS (MDS-UPDRS) or MDS-UPDRSIII are not included.

**Table 2:** Study population, random-effects meta-analyses raw (**A**) and heterogeneity analyses (**B**).

**(A) Raw Analyses**	**PD Patients**	**Controls**	**SMD**	**ci.lb**	**ci.ub**	***p* value**	**I^2^ (%)**	**Tau^2^**	**k**
HF (ms^2^)	145.2 ± 41.1	219.4 ± 48.8	−1.38	−2.17	−0.58	0.002	91	3.27	23
HF (nu)	34.7 ± 1.8	33.2 ± 1.9	0.08	−0.93	1.09	0.867	96	3.99	18
RMSSD (ms)	23.4 ± 1.9	28.9 ± 1.8	−0.58	−1.18	0.02	0.059	92	1.35	18
pNN50 (%)	4.7 ± 1.1	6.8 ± 1.3	−0.46	−1.54	0.63	0.378	96	3.39	14
**(B) Heterogeneity Analyses**	**PD Patients**	**Controls**	**SMD**	**ci.lb**	**ci.ub**	***p* value**	**I^2^ (%)**	**Tau^2^**	**k**
HF (ms^2^)	107.7 ± 11.0	183.0 ± 22.0	−0.79	−1.13	−0.45	<0.001	67	0.28	14
HF (nu)	34.5 ± 3.3	33.8 ± 3.2	0.04	−0.29	0.36	0.810	53	0.12	9
RMSSD (ms)	21.7 ± 1.2	24.7 ± 1.1	−0.65	−0.97	−0.32	0.001	66	0.18	12
pNN50 (%)	3.5 ± 0.5	5.5 ± 0.8	−0.59	−1.06	−0.12	0.020	64	0.28	9

Values are given as mean or as mean ± SD, unless otherwise indicated: (**A**) raw analyses; (**B**) heterogeneity analyses; ci.lb: confidence interval lower bound; ci.ub: confidence interval upper bound; HF: high frequency components of the power spectral density; HRV: heart rate variability; k: number of considered studies; nu: normalized units; PD: Parkinson’s disease; pNN50: number of normal-to-normal intervals differing by more than 50 ms divided by the total number of normal-to-normal intervals; RMSSD: Root mean square of successive normal-to-normal interval differences; SMD: standardized mean differences.

## Data Availability

The data presented in this study are available from the corresponding author on reasonable request.

## Appendix A

**Table A1:** Study selection, characteristics.

Study	Group	HRV Analysis	Measurement	N	Age (y)	N	Age (y)	Disease	Hoehn/Yahr
		Parameter	Duration	(PD)	(PD)	(HC)	(HC)	Duration (y)	Stage
Allan (2006) [18]	PD patients with dementia	HFms^2^	short	40	72	38	76	5.0	na
Arnao (2020), 1 [19]	ambulatory setting, daytime	RMSSD, pNN50	long	18	56	18	56	5.0	na
Arnao (2020), 2 [19]	ambulatory setting, nighttime	RMSSD, pNN50	long	18	56	18	56	5.0	na
Asahina (2014) [20]	PD patients, early untreated	HFms^2^	short	50	64	20	64	1.8	1.6
Barbic (2007), 1 [21]	PD patients without orthostatic hypotension	HFms^2^, HFnu	short	19	66	20	64	7.5	2.7
Barbic (2007), 2 [21]	PD patients with orthostatic hypotension	HFms^2^, HFnu	short	21	69	20	64	10.5	2.8
Bouhaddi (2004), 1 [22]	involvement of L-dopa therapy, newly diagnosed without L-dopa	HFms^2^	short	9	61	9	63	1.2	1.0
Bouhaddi (2004), 2 [22]	involvement of L-dopa therapy, newly diagnosed with L-dopa	HFms^2^	short	9	61	9	63	1.2	1.0
Bouhaddi (2004), 3 [22]	involvement of L-dopa therapy, long-term treated without L-dopa	HFms^2^	short	18	69	9	63	6.0	2.0
Bouhaddi (2004), 4 [22]	involvement of L-dopa therapy, long-term treated with L-dopa	HFms^2^	short	18	69	9	63	6.0	2.0
Brisinda (2013), 1 [23]	PD patients, frequency analysis, during sleep	HFms^2^, HFnu	short	23	63	40	na	na	2.0
Brisinda (2013), 2 [23]	PD patients, frequency analysis, daily activity	HFms^2^, HFnu	short	23	63	40	na	na	2.0
Brisinda (2013), 3 [23]	PD patients, time-domain analysis	RMSSD, pNN50	long	23	63	40	na	na	2.0
Brown (2012), 1 [24]	resting condition, compared to older healthy controls	HFms^2^, HFnu	short	25	na	28	na	na	na
Brown (2012), 2 [24]	deep breathing, compared to older healthy controls	HFms^2^, HFnu	short	25	na	28	na	na	na
Buob (2010) [25]	early stages of PD	RMSSD	long	7	50	7	50	4.0	na
Carricarte (2019), 1 [26]	PD patients, LRRK2-associated	HFms^2^, HFnu, RMSSD	short	14	63	27	59	10.8	na
Carricarte (2019), 2 [26]	PD patients, idiopathic	HFms^2^, HFnu, RMSSD	short	20	64	27	59	6.3	na
Delgado (2014), 1 [27]	Mexican PD patients, supine resting	HFms^2^, HFnu, RMSSD, pNN50	short	20	61	20	38	3.7	na
Delgado (2014), 2 [27]	Mexican PD patients, active standing	HFms^2^, HFnu, RMSSD, pNN50	short	20	61	20	38	3.7	na
Delgado (2014), 3 [27]	Mexican PD patients, controlled breathing	HFms^2^, HFnu, RMSSD, pNN50	short	20	61	20	38	3.7	na
Devos (2003), 1 [28]	untreated PD patients, disease duration less than 2 years, daytime	HFms^2^	long	10	60	10	61	1.5	na
Devos (2003), 2 [28]	untreated PD patients, disease duration less than 2 years, nighttime	HFms^2^, pNN50	long	10	60	10	61	1.5	na
Devos (2003), 3 [28]	treated PD patients, disease duration more than 2 years, daytime	HFms^2^	long	10	63	10	61	8.0	na
Devos (2003), 4 [28]	treated PD patients, disease duration more than 2 years, nighttime	HFms^2^, pNN50	long	10	63	10	61	8.0	na
Devos (2003), 5 [28]	treated PD patients, advanced PD with motor complications, daytime	HFms^2^	long	10	62	10	61	8.6	na
Devos (2003), 6 [28]	treated PD patients, advanced PD with motor complications, nighttime	HFms^2^, pNN50	long	10	62	10	61	8.6	na
Gjerloff (2015) [29]	association with Donepezil positron emission tomography	HFms^2^, RMSSD	short	12	64	12	62	5.3	2.2
Haapaniemi (2001) [30]	ambulatory setting, 24 h	HFms^2^	long	54	61	47	60	1.7	1.5 #
Harnod (2014) [31]	association with motor symptom duration	HF (ln) *	short	32	62	32	na	9.8	2.7
Jain (2011) [32]	association with pupil measures	HF (*10^−1^s^2^/Hz) *	short	17	65	18	60	na	1.7
Jaipurkar (2018), 1 [33]	PD patients, supine resting	HFms^2^ *	short	31	61	31	60	3.6	na
Jaipurkar (2018), 2 [33]	PD patients, head-up tilt table test	HFms^2^ *	short	31	61	31	60	3.6	na
Kallio (2000), 1 [34]	PD patients, untreated	RMSSD	short	50	60	55	56	2.2	1.7
Kallio (2000), 2 [34]	PD patients, untreated, fast Fourier transform analysis	HFnu, pNN50	short	20	na	24	na	na	na
Kallio (2002) [35]	PD patients, untreated, fast Fourier transform analysis	HFms^2^, HFnu	short	32	58	24	54	na	na
Kallio (2004), 1 [36]	association with nocturnal sleep patterns, awake	HFms^2^, HFnu	long	21	58	22	56	1.8	1.5
Kallio (2004), 2 [36]	association with nocturnal sleep patterns, REM	HFms^2^, HFnu	long	21	58	22	56	1.8	1.5
Kallio (2004), 3 [36]	association with nocturnal sleep patterns, sleep stage 1	HFms^2^, HFnu	long	21	58	22	56	1.8	1.5
Kallio (2004), 4 [36]	association with nocturnal sleep patterns, sleep stage 2	HFms^2^, HFnu	long	21	58	22	56	1.8	1.5
Kallio (2004), 5 [36]	association with nocturnal sleep patterns, sleep stage 3	HFms^2^, HFnu	long	21	58	22	56	1.8	1.5
Kallio (2004), 6 [36]	association with nocturnal sleep patterns, sleep stage 4	HFms^2^, HFnu	long	21	58	22	56	1.8	1.5
Kanegusuku (2017), 1 [37]	effect of progressive resistance training, PD training group	HFnu	short	15	67	16	68	8.5	2.5
Kanegusuku (2017), 2 [37]	effect of progressive resistance training, PD control group	HFnu	short	12	63	16	68	9.0	2.4
Kang (2012) [38]	association with olfactory dysfunction	HFms^2^ *	short	15	66	18	60	na	1.7
Katagiri (2015) [39]	association with myocardial scintigraphy	HFms^2^	short	50	66	50	67	na	na
Ke (2017) [40]	association with sympathetic skin response	HFms^2^, RMSSD, pNN50	long	48	69	30	63	5.4	na
Kim (2016), 1 [41]	PD patients, mild stage	RMSSD, pNN50	long	106	66	25	67	2.2	1.1
Kim (2016), 2 [41]	PD patients, moderate stage	RMSSD, pNN50	long	51	72	25	67	4.5	2.2
Kim (2016), 3 [41]	PD patients, severe stage	RMSSD, pNN50	long	31	71	25	67	5.8	3.2
Kiyono (2012) [42]	ambulatory setting, daytime	HF (ln) *, RMSSD	long	10	69	60	69	10.7	3.6
Liou (2013), 1 [43]	association with electroencephalography, quiet breathing	HFnu	short	26	67	23	65	2.6	1.3
Liou (2013), 2 [43]	association with electroencephalography, deep breathing	HFnu	short	26	67	23	65	2.6	1.3
Maetzler (2015) [44]	association with sympathetic skin response, metronomic breathing	HFnu, RMSSD, pNN50	short	45	66	26	65	3.8	2.1
Meco (2000), 1 [45]	effect of treatment with Tolcapone (before treatment), daytime	HFms^2^	long	7	70	7	na	14.1	2.1
Meco (2000), 2 [45]	effect of treatment with Tolcapone (before treatment), nighttime	HFms^2^	long	7	70	7	na	14.1	2.1
Mihci (2006) [46]	ambulatory setting, 24 h	RMSSD, pNN50	long	23	66	15	67	5.5	2.5 #
Niwa (2011), 1 [47]	PD patients, early stage, daytime	HFms^2^	long	9	71	30	69	4.8	na
Niwa (2011), 2 [47]	PD patients, early stage, nighttime	HFms^2^	long	9	71	30	69	4.8	na
Niwa (2011), 3 [47]	PD patients, advanced stage, daytime	HFms^2^	long	18	69	30	69	7.1	na
Niwa (2011), 4 [47]	PD patients, advanced stage, nighttime	HFms^2^	long	18	69	30	69	7.1	na
Pursiainen (2002), 1 [48]	PD patients, untreated, daytime	HFms^2^	long	44	63	43	60	1.7	1.8
Pursiainen (2002), 2 [48]	PD patients, untreated, nighttime	HFms^2^	long	44	63	43	60	1.7	1.8
Pyatigorskaya (2016), 1 [49]	association with magnetic resonance imaging, slow wave sleep	HFnu	long	47	62	23	60	na	2.0
Pyatigorskaya (2016), 2 [49]	association with magnetic resonance imaging, REM sleep	HFnu	long	47	62	23	60	na	2.0
Rocchi (2018) [50]	comparison to second control group (REM sleep behavior disorder)	HFnu	short	17	68	12	69	2.3	na
Rocha (2018) [51]	effect of game therapy training (before training)	HFnu, RMSSD	short	31	78	40	72	8.0	2.0
Sauvageot (2011), 1 [52]	association with nocturnal sleep patterns, Non REM	HFnu, pNN50	long	35	66	35	65	6.6	2.4
Sauvageot (2011), 2 [52]	association with nocturnal sleep patterns, REM	HFnu, pNN50	long	35	66	35	65	6.6	2.4
Solla (2015), 1 [53]	PD patients, tremor dominant subtype	HFnu, RMSSD, pNN50	long	17	63	17	65	6.0	2.1
Solla (2015), 2 [53]	PD patients, akinetic-rigid subtype	HFnu, RMSSD, pNN50	long	11	66	17	65	7.9	2.7
Sorensen (2013), 1 [54]	association with rapid-eye movement sleep behavior, awake	HFms^2^, HFnu, RMSSD, pNN50	short	10	63	10	59	na	1.4
Sorensen (2013), 2 [54]	association without rapid-eye movement sleep behavior, awake	HFms^2^, HFnu, RMSSD, pNN50	short	13	61	10	59	na	0.9
Sorensen (2013), 3 [54]	association with rapid-eye movement sleep behavior, Non REM	HFms^2^, HFnu, RMSSD, pNN50	short	10	63	10	59	na	1.4
Sorensen (2013), 4 [54]	association without rapid-eye movement sleep behavior, Non REM	HFms^2^, HFnu, RMSSD, pNN50	short	13	61	10	59	na	0.9
Sorensen (2013), 5 [54]	association with rapid-eye movement sleep behavior, REM	HFms^2^, HFnu, RMSSD, pNN50	short	10	63	10	59	na	1.4
Sorensen (2013), 6 [54]	association without rapid-eye movement sleep behavior, REM	HFms^2^, HFnu, RMSSD, pNN50	short	13	61	10	59	na	0.9
Sriranjini (2011) [55]	effect of a single dose L-dopa (before intake)	HFms^2^, HFnu, RMSSD, pNN50	short	11	57	11	55	4.1	2.1
Sumi (2012), 1 [56]	effect of deep brain stimulation (before stimulation), off medication	HFms^2^	short	28	62	13	58	22.0	3.9
Sumi (2012), 2 [56]	effect of deep brain stimulation (before stimulation), on medication	HFms^2^	short	28	62	13	58	22.0	2.4
Szili-Török (1999), 1 [57]	association with baroreflex sensitivity, normal	HF (ms/Hz) *, RMSSD, pNN50	short	12	64	18	65	na	2.0
Szili-Török (1999), 2 [57]	association with baroreflex sensitivity, impaired	HF (ms/Hz) *, RMSSD, pNN50	short	8	67	18	65	na	2.1
Trachani (2012) [58]	effect of deep brain stimulation (before stimulation)	HFms^2^ *, HFnu *	short	24	62	24	na	12.8	3.1
Valenza (2016) [59]	computational assessment of heartbeat dynamics	HFms^2^	short	30	67	29	61	na	na
Visanji (2017), 1 [60]	PD patients, LRRK2-associated	HF (log) *, RMSSD *	short	20	64	32	59	11.5	na
Visanji (2017), 2 [60]	PD patients, idiopathic	HF (log) *, RMSSD *	short	26	64	32	59	6.2	na
Walter (2018) [61]	association with vagus nerve atrophy	RMSSD *	short	20	73	20	70	10.1	na
Weise (2015) [62]	association with auricular branch of vagus nerve stimulation	HF (ln) *	short	50	64	50	64	6.4	2.3
Yoon (2016) [63]	PD patients, tremor dominant subtype, drug naiv	HFms^2^, RMSSD	short	27	64	23	63	1.6	na
Zawadka-Kunikowska (2017), 1 [64]	association with peripheral vascular resistance, vasodilation reaction	HFms^2^	short	15	67	47	66	9.0	2.9
Zawadka-Kunikowska (2017), 2 [64]	association with peripheral vascular resistance, vasoconstriction reaction	HFms^2^	short	41	69	47	66	6.0	2.0

Values are given as the mean, unless otherwise indicated: #: values are given as the median; na: nonreported data; *: not considered in further analyses because data are not plausible or reported in rarely used units; HC: healthy controls; HF: high-frequency components of the power spectral density; nu: normalized units; PD: Parkinson’s disease; pNN50: number of normal-to-normal intervals differing by more than 50 ms divided by the total number of normal-to-normal intervals; RMSSD: root mean square of successive normal-to-normal interval difference.
